# Beneficial Effects of Indigenous Probiotics in High-Cholesterol Diet-Induced Hypercholesterolemic Rats

**DOI:** 10.3390/nu15122710

**Published:** 2023-06-11

**Authors:** Narathip Puttarat, Anongnard Kasorn, Porntipha Vitheejongjaroen, Chantanapa Chantarangkul, Marut Tangwattanachuleeporn, Malai Taweechotipatr

**Affiliations:** 1 Center of Excellence in Probiotics, Srinakharinwirot University, Bangkok 10110, Thailand; 2 Department of Microbiology, Faculty of Medicine, Srinakharinwirot University, Bangkok 10110, Thailand; 3 Department of Biomedical Science, Faculty of Medicine, Vajira Hospital, Navamindradhiraj University, Bangkok 10300, Thailand; 4 Faculty of Allied Health Sciences, Burapha University, Chonburi 20131, Thailand; 5 Research Unit for Sensor Innovation (RUSI), Burapha University, Chonburi 20131, Thailand

**Keywords:** probiotics, bile salt hydrolase, cholesterol-lowering activity, hypercholesterolemia, microbiota

## Abstract

Hypercholesterolemia is a significant risk factor for cardiovascular disease and metabolic disorders. Probiotics are the essential constituents of the gastrointestinal microbiota that provide health-promoting effects. Cholesterol-lowering activity is a specific property of probiotics, improving the cholesterol metabolism without adverse effects. Thus, the purpose of this study was to investigate the hypocholesterolemic effect of single and mixed cholesterol-lowering probiotic strains (including *Limosilactobacillus reuteri* TF-7, *Enterococcus faecium* TF-18, and *Bifidobacterium animalis* TA-1) in high-cholesterol diet (HCD)-induced hypercholesterolemic rats. The results showed that the administration of single probiotics contributed to a reduction in the body weight gain, visceral organ indexes, hyperlipidemia, and hepatic steatosis and also an improvement in the gastrointestinal microbiota. Besides the effect of single cholesterol-lowering probiotics, three probiotics strains could also synergize their hypocholesterolemic effect when administered simultaneously. These findings indicate that three cholesterol-lowering probiotic strains are suitable for development as probiotic supplements to reduce the risk of diseases caused by cholesterol and exert health benefits with synergistic effect when administered simultaneously.

## 1. Introduction

Hypercholesterolemia, which is a form of hyperlipidemia, is estimated to cause approximately 40% of deaths by 2030 [1,2]. Hypercholesterolemia is mostly a consequence of an acquired trait by the consumption of a high-fat diet. It is characterized by the presence of elevated lipid profiles, including total cholesterol (TC), triacylglycerol (TG), and low-density lipoprotein cholesterol (LDL-C). However, the level of high-density lipoprotein cholesterol (HDL-C) is low in this condition [3]. Although hypocholesterolemic drugs are available for the treatment of high cholesterol, their various adverse effects, such as myopathy and rhabdomyolysis, have been reported [4]. Therefore, prevention and treatment of hypercholesterolemia without adverse effects are important challenges in medical strategy.

Probiotics are defined as “live microorganisms that, when administered in adequate amounts, confer a health benefit on the host” [5]. Probiotics can produce various beneficial health effects with host–microbe interactions that promote the metabolic homeostasis in the gastrointestinal system [6]. Previous studies have reported the use of probiotics to reduce hypercholesterolemia and modulate the gastrointestinal microbiota [7]. Bile salt hydrolase (BSH) enzyme activity and cholesterol assimilation are the major mechanisms of probiotics to reduce cholesterol levels [8]. Certain strains of probiotics secrete BSH, which converts conjugated bile salts into deconjugated bile salts in the human gastrointestinal tract, that can cause a reduction in lipid emulsification and absorption in the intestine [9,10]. Moreover, biodegradation of bile salts by BSH stimulates cholesterol metabolism in liver. Thus, the liver synthesizes new bile salts through the mevalonate pathway, resulting in a reduction in cholesterol levels and modulation of liver function in lipid metabolism [11]. In addition, probiotic cells also incorporate cholesterol into plasma membrane, a mechanism called cholesterol assimilation, to further reduce the cholesterol level [12]. Therefore, probiotics with cholesterol-lowering activity expressed in gastrointestinal tract is a suitable alternative treatment for hypercholesterolemia with a long-lasting protective effect.

Probiotics can be obtained by food consumption. Consuming a variety of products with probiotics can promote diversity of gastrointestinal microbiota, which plays a significant role in health-promoting effects [13]. Each probiotic genus, especially *Lactobacillus*, *Enterococcus*, *Bifidobacterium*, and *Streptococcus,* residng in the gastrointestinal microbiota strikingly exerts different functional characteristics [14]. According to the synergistic effect hypothesis, it is a crucial criterion to select different genera of cholesterol-lowering probiotics to evaluate the synergistic effect of cholesterol reduction for further development as cholesterol-lowering probiotic supplements. Our previous study identified different genera of probiotics with the most robust cholesterol reduction property, including *Limosilactobacillus reuteri* TF-7 (formerly named as *Lactobacillus reuteri* TF-7), *E. faecium* TF-18, and *B. animalis* TA-1. They also possessed other probiotic characteristics. Thus, with their properties, these probiotics are suitable to be applied in this study [15]. Therefore, the purpose of this study was to investigate hypocholesterolemic effect of single and mixed probiotic strains on body weight gain, visceral organ indexes, lipid profiles, hepatic steatosis, and fecal microbiota in high-cholesterol diet (HCD)-induced hypercholesterolemic rats.

## 2. Materials and Methods

### 2.1. Probiotic Strains and Culture Condition

Three cholesterol-lowering probiotics, originally isolated from traditional Thai foods, were cultured in de Man, Rogosa, and Sharpe (MRS) agar (Himedia, Mumbai, India) under anaerobic conditions at 37 °C for 48 h. A single colony of probiotics was picked up to culture in MRS broth and then incubated at the same conditions. After that, bacterial cells were harvested using centrifugation at 5000× *g*, 4 °C for 10 min and diluted in sterile phosphate-buffered saline (PBS, 0.1 M, pH 7.2) to a concentration of 10^9^ colony forming unit (CFU)/mL.

### 2.2. Animal Housing and Feeding

Thirty-five male Wistar rats (aged 8 weeks with 120–140 g weight) were purchased from the Nomura Siam International (Bangkok, Thailand). All animal procedures were performed according to the guidelines of the Ethics and Research Standardization Section, Srinakharinwirot University (approval number: COA/AE-013-2562). All rats were randomly divided into 7 groups of 5 individuals and were housed in plastic cages with wire mesh clover in room conditions at a temperature of 24 ± 1 °C, 12/12 h light-dark cycle, and 60 ± 5% relative humidity. The acclimatization was performed prior to experiments for a week on a basal diet (082G/15, National Laboratory Animal Center, Mahidol University, Bangkok, Thailand) containing 52% (*w*/*w*) carbohydrate, 24% (*w*/*w*) protein, 4.5% (*w*/*w*) fat, 5% (*w*/*w*) fiber, 0.9% (*w*/*w*) phosphorus, 1% (*w*/*w*) calcium, and 12.6% (*w*/*w*) moisture. All rats were then randomly assigned to seven groups with similar average body weight. All groups were fed with basal diet, had free water access (ad libitum), and received different supplements by daily oral gavage for eight weeks as follows:

(Control), control group received 2 mL of PBS;
(Probiotics), probiotics control group received 1 mL of 1 × 10^9^ CFU/mL mixed three probiotic strains and 1 mL of PBS;
(HCD), HCD group received 1 mL of HCD and 1 mL of PBS;
(HCD-L), HCD supplemented with *L. reuteri* TF-7 group received 1 mL of HCD and 1 mL of 1 × 10^9^ CFU/mL *L. reuteri* TF-7;
(HCD-E), HCD supplemented with *E. faecium* TF-18 group received 1 mL of HCD and 1 mL of 1 × 10^9^ CFU/mL *E. faecium* TF-18;
(HCD-B), HCD supplemented with *B. animalis* TA-1 group received 1 mL of HCD and 1 mL of 1 × 10^9^ CFU/mL *B. animalis* TA-1;
(HCD-mix), HCD supplemented with mixed three probiotic strains group received 1 mL of HCD and 1 mL of 1 × 10^9^ CFU/mL mixed three probiotic strains.

HCD was prepared based on Paigen atherogenic diet with minor modifications, i.e., 1.25% (*w*/*v*) cholesterol, 0.5% (*w*/*v*) sodium cholate, 12.5% (*w*/*v*) sucrose, and 17.2% (*w*/*v*) fat as the major constituents [16]. Body weight of rats was measured every week, and daily food intake was recorded in the morning.

### 2.3. Sample Collection

Feces of rats were collected at the end of the experiment to analyze the fecal microbiota. At the end of the experiment, all rats were made to fast for 10 h prior to euthanasia. Then, they were sacrificed to collect blood using cardiac puncture. Abdominal fat and liver were harvested, rinsed, and weighed after blotting them dry. After that, the liver was fixed with 10% (*v*/*v*) formaldehyde.

### 2.4. Biochemical Analysis

Plasma was collected using centrifugation of heparinized blood at 1500× *g*, 25 °C for 15 min. Biochemical parameters, including TC, TG, HDL-C, LDL-C, aspartate transaminase (AST), and alanine transaminase (ALT), were examined in the plasma by the Professional Laboratory Management Corp, Co., Ltd. (Bangkok, Thailand).

### 2.5. Liver Histological Analysis

Right lobe of a rat’s liver was sectioned and embedded in paraffin. Liver tissue sections with a thickness of 5 μm were stained with hematoxylin and eosin (H&E) and Masson’s trichome. Immunohistochemical staining for F4/80 surface molecules of macrophages was performed.

For Masson’s trichrome staining, liver tissue sections were deparaffinized and rehydrated using gradient alcohol. Then, re-fixed in Bouin’s solution for 1 h at 56 °C, rinsed with running tap water for 5 min, and stained in Weigert’s iron hematoxylin for 5 min. The sections were rinsed with running tap water again and stained in Biebrich’s scarlet-acid fuchsin solution for 20 min and washed in distilled water. After that, the sections were differentiated in phosphotungstic-phosphomolybdic acid for 10 min, transferred to aniline blue solution for 8 min, rinsed briefly in distilled water, and dipped in 1% acetic acid for 1 time and then washed again in distilled water and mounted on slides.

For F4/80 immunohistochemical staining, the paraffin-embedded liver tissues were section at 5 μm, mounted on charged glass slides, deparaffinized in xylene, and stained with F4/80 antibody for 32 min. Then, the tissues were counterstained briefly using one drop of hematoxylin and one drop of bluing reagent. The histology of liver tissues was studied using light microscopy at an original magnification of 400× [17].

### 2.6. Fecal Microbiota Analysis

Microbial DNA was extracted from 200 μg of feces using QIAamp DNA Stool Mini Kit (Qiagen, Germantown, MD, USA) according to the manufacturer’s instruction. After extraction, DNA concentration was determined using NanoDrop One (Thermo Fisher Scientific, Waltham, MA, USA). Sequencing of 16 s rRNA of microbiota were performed using next-generation sequencing (Getz Healthcare Ltd., Bangkok, Thailand). The fecal microbiota changes from different groups were analyzed and expressed as a heatmap correlation [18].

### 2.7. Statistical Analysis

All experiments were performed three times independently. The results were expressed as means ± standard deviation (SD). Statistically significant analysis of data was performed with ANOVA with Dunnett’s or Tukey’s multiple comparison test using the GraphPad Prism software (GraphPad software, version 8.0, San Diego, CA, USA). A *p* value less than 0.05 was considered significant.

## 3. Results

### 3.1. Effects of Cholesterol-Lowering Probiotics on Body Weight Gain, Food Intake, Food Efficiency, and Visceral Organ Indexes of Rats

Individual and synergistic cholesterol-lowering effects of *L. reuteri* TF-7, *E. faecium* TF-18, and *B. animalis* TA-1 were investigated in Wistar rat model. During the experimental period, body weight of rats in all groups increased with the highest body weight gain observed in the HCD group (Table 1). Although there was no statistically significant difference in body weight gain among HCD-fed groups supplemented with single or mixed cholesterol-lowering probiotic strains (HCD-L, HCD-E, HCD-B, and HCD-mix groups), their body weight gains were higher than C and P groups with statistical significance (*p* < 0.05). Interestingly, the body weight gains were significantly reduced in all treated groups after administration of probiotics for 8 weeks (*p* < 0.05) when compared with the HCD group as shown in Table 1.

Considering the result in Table 2, there was no difference in food intake among the seven groups. However, statistically significant higher food efficiency of HCD, HCD-L, HCD-E, and HCD-B groups was observed when compared with control and probiotics groups (Table 3). Moreover, the abdominal fat and liver weights were measured to analyze the visceral organ indexes. The results of abdominal fat and liver indexes in each group were consistent with their body weight gain, and the reduction of visceral organ indexes was found in HCD-L, HCD-E, HCD-B, and HCD-mix groups compared with control, probiotics, and HCD groups (Table 4).

### 3.2. Effects of Cholesterol-Lowering Probiotics on Lipid Profiles of Rats

**Figure 1:**
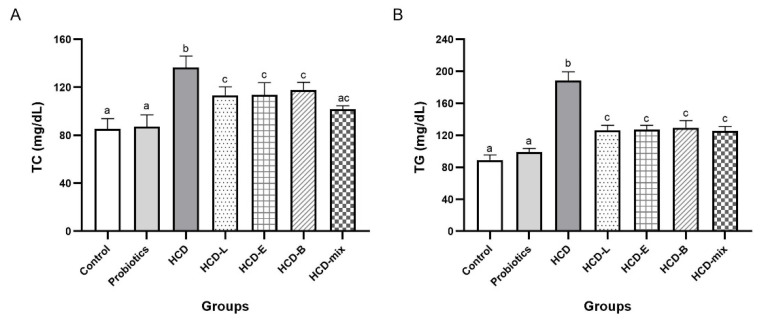

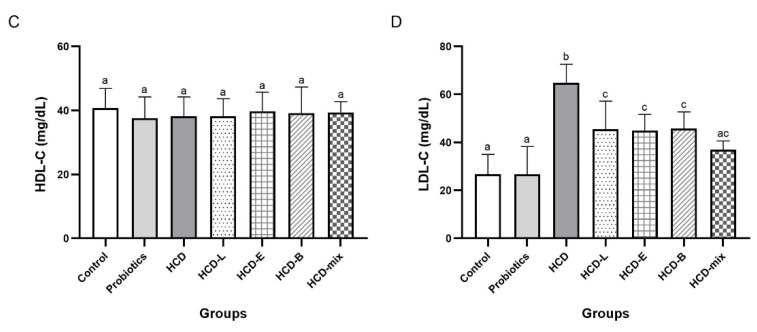
Effect of cholesterol-lowering probiotic administrations on levels of lipid profile in HCD-induced hypercholesterolemic rats at the end of the experiment. (**A**) total cholesterol (TC), (**B**) triglyceride (TG), (**C**) high-density lipoprotein cholesterol (HDL-C), and (**D**) low-density lipoprotein cholesterol (LDL-C). Error bars represent standard deviations of means (n = 5). The different lowercase letters a–c in each index indicate statistically significant differences (*p* < 0.05).

To evaluate whether single and mixed cholesterol-lowering probiotic administrations can reduce hyperlipidemia in HCD-fed rats, the levels of TC, TG, HDL-C, and LDL-C were measured. Based on the result in Figure 1, TC, TG, and LDL-C levels of HCD group significantly increased when compared with other groups (*p* < 0.05). Similar levels of TC, TG, and LDL-C among HCD-L, HCD-E, HCD-B, and HCD-mix groups were observed. These results were significantly lower when compared with HCD group (*p* < 0.05). Interestingly, administration of mixed cholesterol-lowering probiotics (HCD-mix group) reduced TC and LDL-C levels to levels similar to those in normal diet-fed rats in both control and probiotics groups. However, there was no statistical difference in HDL-C levels in all experimental groups.

### 3.3. Effects of Cholesterol-Lowering Probiotics on Hepatic Steatosis of Rats

**Figure 2:**
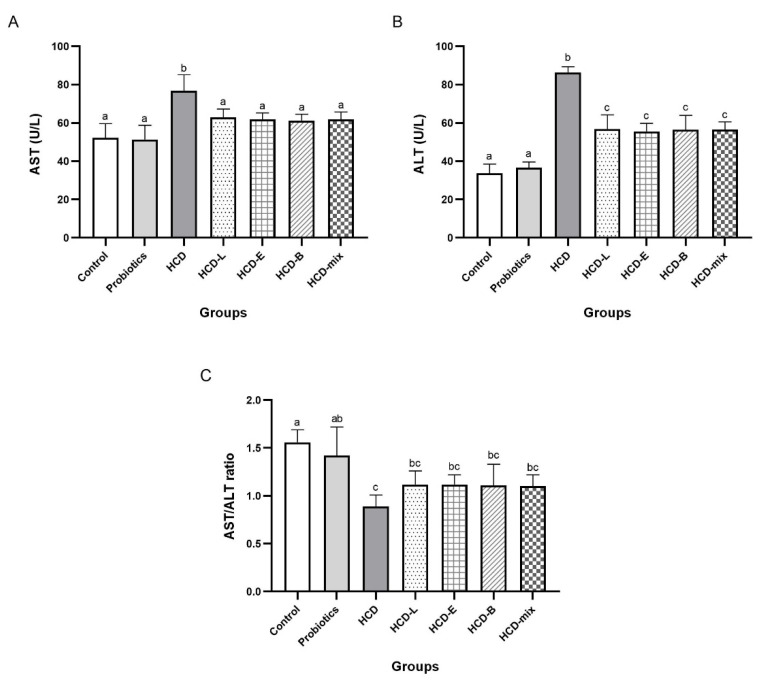
Effect of cholesterol-lowering probiotic administrations on levels of (**A**) aspartate aminotransferase (AST), (**B**) alanine aminotransferase (ALT), and (**C**) AST/ALT ratio in HCD-induced hypercholesterolemic rats at the end of the experiment. Error bars represent standard deviations of means (n = 5). The different lowercase letters a–c in each index indicate statistically significant differences (*p <* 0.05).

The predominant liver enzymes, including AST and ALT, are the important parameters for indicating liver injury. Thus, AST and ALT levels were measured in combination with the result of liver histological analysis to determine the hypocholesterolemic effect on HCD-induced hepatic steatosis. Significant elevation of AST and ALT levels and a reduction of AST/ALT ratio were observed in HCD group as shown in Figure 2A,B. In contrast, AST and ALT levels in HCD-L, HCD-E, HCD-B, and HCD-mix groups were significantly reduced group (*p* < 0.05) with an increase in AST/ALT ratio, which was consistent with control and probiotics groups (Figure 2C).

**Figure 3:**
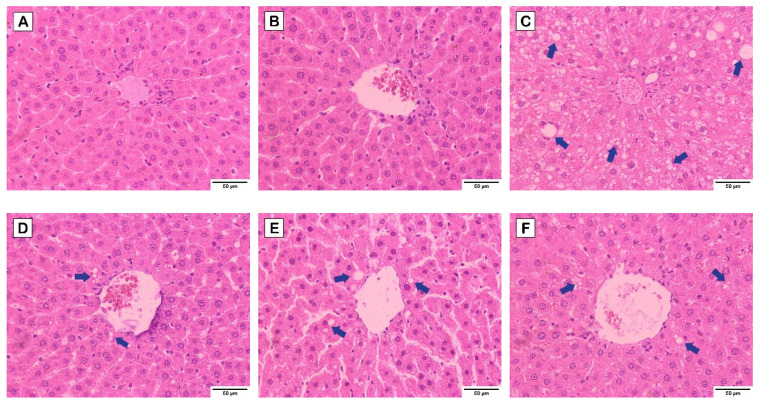

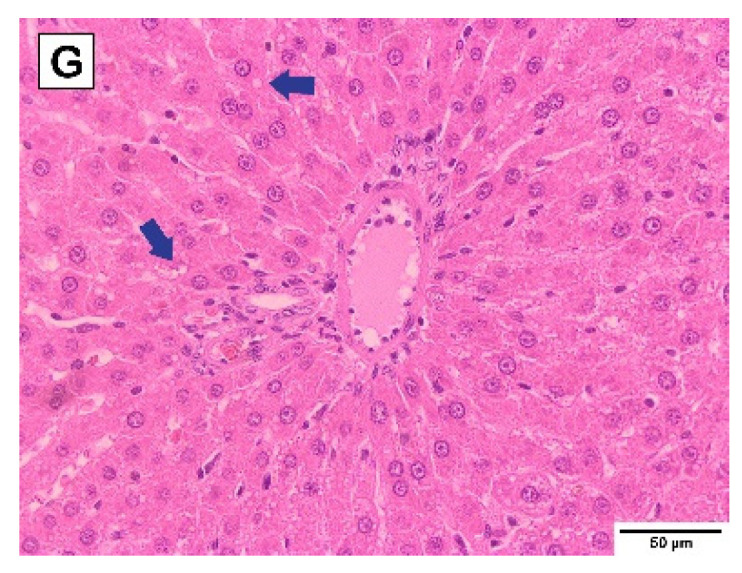
H&E staining of liver tissues (magnification of 400×) of high-cholesterol diet-induced hypercholesterolemic rats: (**A**) control, (**B**) probiotics, (**C**) HCD, (**D**) HCD-L, (**E**) HCD-E, (**F**) HCD-B, and (**G**) HCD-mix groups. The blue arrow head indicates lipid droplets.

**Figure 4:**
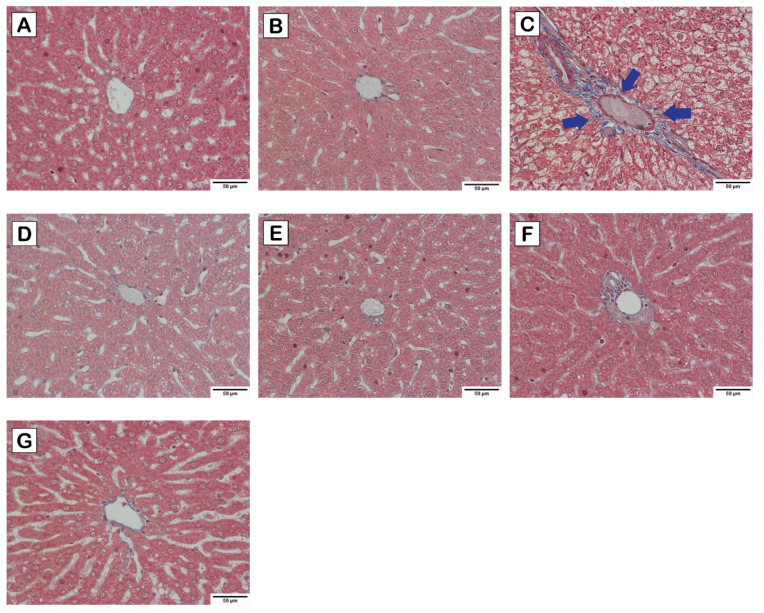
Masson’s trichrome staining of liver tissue sections (magnification of 400×) of high-cholesterol diet-induced hypercholesterolemic rats: (**A**) control, (**B**) probiotics, (**C**) HCD, (**D**) HCD-L, (**E**) HCD-E, (**F**) HCD-B, and (**G**) HCD-mix groups. The blue arrow head indicates collagen fibers.

**Figure 5:**
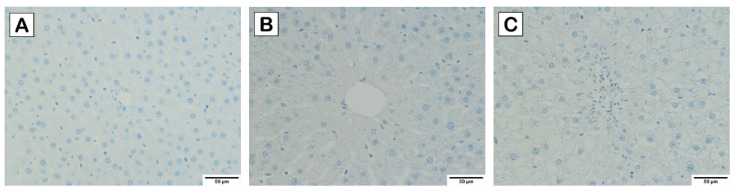

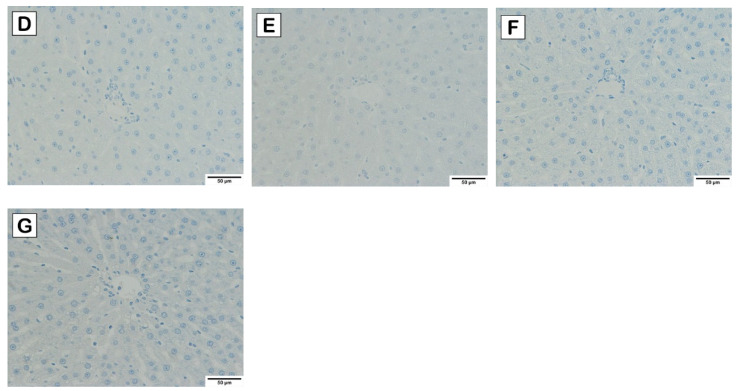
F4/80 immunohistochemical staining of liver tissues (magnification of 400×) of diet-induced hypercholesterolemic rats: (**A**) control, (**B**) probiotics, (**C**) HCD, (**D**) HCD-L, (**E**)HCD-E, (**F**) HCD-B, and (**G**) HCD-mix groups.

Based on the liver histology as illustrated in Figure 3, Figure 4 and Figure 5, normal hepatocytes were found in control and probiotics groups, in which the hepatic cells were stained by H&E (Figure 3), and clearly showed acidophilic and basophilic regions of cytoplasm and nucleus, respectively. Their microscopic structure appeared round shaped with a clear boundary, and the nucleus occupied the center of the cells. In contrast, increasing vacuolization of lipid droplets extensively disseminated and accumulated in the hepatocytes of HCD group. Notably, the hepatocytes appearance in HCD-L, HCD-E, HCD-B, and HCD-mix groups showed a lower degree of vacuolization than the HCD group. Furthermore, the perisinusoidal space was similar to the normal hepatocytes. Similarly, this is seen in Masson’s trichrome staining (Figure 4), which is used to indicate collagen fibers that develop into fibrosis. The collagen fibers were absent in the liver tissues of the control and probiotics groups (Figure 4A,B) as well as in the probiotic-treated group (Figure 4D–G). In HCD group, fibrosis was initiated with collagen fiber aggregation in the pericentral and periportal areas (blue arrow) (Figure 4C). In addition, F4/80 immunohistochemical staining was performed to detect M1-macrophages (bone marrow-derived macrophages) infiltrating the liver. Similarly, the liver residential macrophages were also stained. As shown in Figure 5, an increase of F4/80-positive cells was not observed in the liver tissues of probiotics, HCD, HCD-L, HCD-E, HCD-B, and HCD-mix groups compared with the control group.

### 3.4. Effects of Cholesterol-Lowering Probiotics on Modulating Fecal Microbiota of Rats

**Figure 6:**
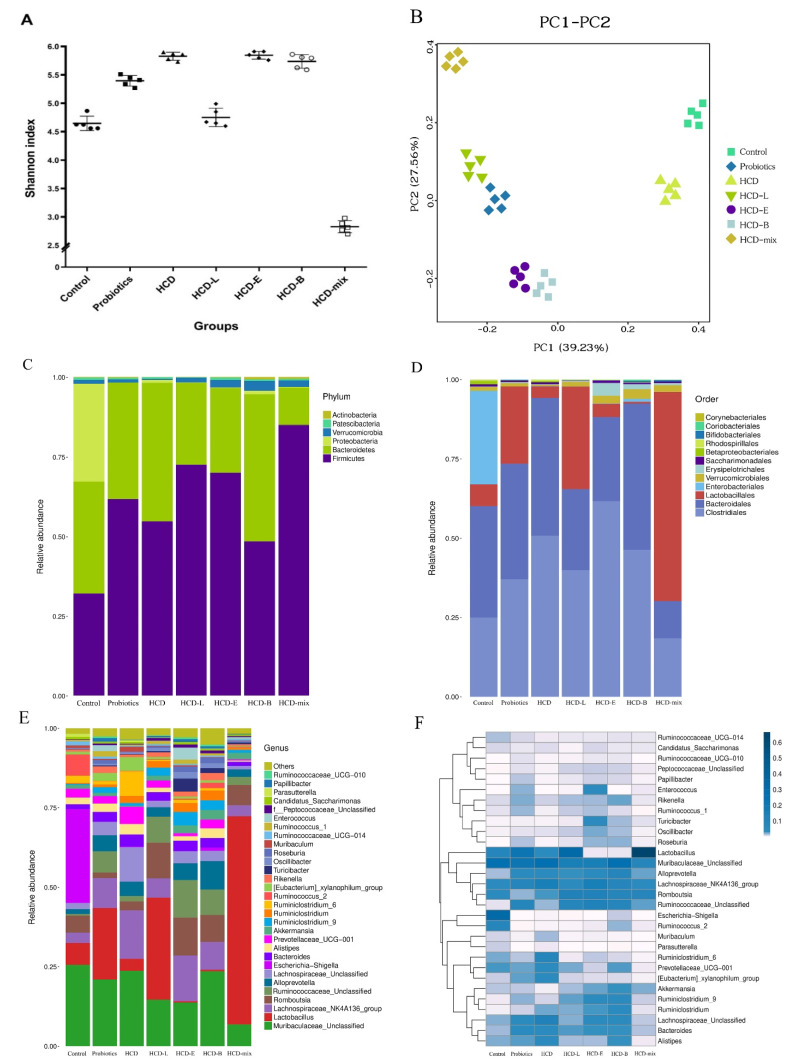
Effect of probiotic administrations on composition of gut microbiota in HCD-induced hypercholesterolemic rats at the end of the experiment. (**A**) Alpha diversity indices, Shannon, (**B**) beta diversity, PCoA score plots, (**C**) the relative abundance of gut microbiota in the phylum, (**D**) order, (**E**) genus level, and (**F**) heatmap of the gut microbiota at the genus level. All data are indicated as mean of five rats per group.

The fecal microbiota diversity was determined to prove the modulation of fecal microbiota after oral administration of probiotics. In alpha diversity (Figure 6A), the Shannon diversity index of probiotic groups was significantly different when compared with other groups. Moreover, there was no statistical difference in microbiota diversity when HCD group was compared with HCD-E and HCD-B groups. Although the Shannon index of HCD-L group was similar to the control, it was lower than HCD, HCD-E, and HCD-B groups. In addition, the lowest Shannon index at 2.828 was observed in HCD-mix group. Beta diversity was also assessed in different cohorts. Scatter-based plot based on PcoA scores clearly showed a constituent community split between the seven groups. PC1 and PC2 were 39.23% and 27.56% of the total variance, respectively (Figure 6B). As shown in Figure 6C, the most abundant bacteria in the feces belonged to the phyla *Firmicutes*, *Bacteroidetes*, and *Proteobacteria. Bacteroidales*, *Clostridiales*, and *Enterobacteriales* dominated in the feces of normal rats. Consistently, a significant reduction in *Lactobacillales* abundance and a higher abundance of *Clostridiales*, *Bacteroidales*, and *Verrucomicrobiales* were observed in HCD rats (Figure 6D). The higher numbers of probiotic genera, including *Lactobacillus*, *Bifidobacterium*, *Enterococcus, Akkermansia*, and *Ruminococcaceae*, were observed in HCD-L, HCD-E, HCD-B, and HCD-mix groups when compared with HCD group (Figure 6E,F). Furthermore, decreases in number of *Lachnospiraceae, Bacteroides, Alistipes*, *and Prevotellaceae* were observed in these rats after oral administration of probiotics.

## 4. Discussion

Hypercholesterolemia is a causative factor for metabolic disorders. Thus, the functional properties of probiotics have gained a lot of attention due to their beneficial health effects [19]. Recently, several studies have demonstrated that cholesterol-lowering activity of probiotics could ameliorate hypercholesterolemia [8]. Moreover, a meta-analysis elucidated that oral administration of mixed probiotic strains exhibited synergistic effects in reducing lipid levels and enhancing liver function in hypercholesterolemic rats [18]. Therefore, three indigenous probiotics with BSH activity and cholesterol assimilation were used in this study to evaluate their cholesterol-lowering activity in HCD-fed rats.

As expected, our results showed significant elevation in body weight gain, abdominal fat and liver indexes (Table 1, Table 2, Table 3 and Table 4), lipid levels, and hepatic steatosis after feeding rats with HCD (Figure 1). The major composition of HCD is fat that induces hypercholesterolemia and other pathologic effects. Oral administration of three cholesterol-lowering probiotic strains for eight weeks significantly decreased body weight gain, abdominal fat, lipid levels, liver indexes, and hepatic steatosis as well as improved the fecal microbiota. These results were in agreement with a previous study that reported the supplementation of *L. reuteri* and *Lactiplantibacillus plantarum* (*L. plantarum*) reduced lipid profiles and liver injury by modulating the mevalonate pathway in hypercholesterolemic rats [20]. Considering the result of lipid profiles, the hypocholesterolemic effect of three probiotic strains showed a significant reduction in TC, TG, and LDL-C levels. Interestingly, the mixed cholesterol-lowering probiotic strains exhibited synergistic effect and reduced TC and LDL-C levels similar to normal diet-fed rats. However, HDL-C levels were not suppressed. This might be due to the short experimental duration or low efficiency of probiotics to stimulate an increase of HDL-C [21,22]. Excess cholesterol is transported by LDL-C and deposited in blood vessels that eventually contributes to the buildup of atheromatous plaque. Hypertriglyceridemia also increases a risk for metabolic disorders, stroke, high blood pressure, and heart attack [23]. Thus, these lipid profile parameters are strong indicators of cardiovascular disease and metabolic disorders.

Excess cholesterol is also accumulated in hepatic cells, leading to non-alcoholic fatty liver disease (NAFLD) [1,24]. Therefore, pathology of liver was examined to demonstrate the hypocholesterolemic effect of probiotics on HCD-induced hepatic steatosis. AST and ALT enzymes are abundantly contained in the hepatocytes and released in the blood circulation after liver damage [25]. Thus, liver injury is characterized by an increase in AST and ALT levels and a decrease in AST/ALT ratio. From the results (Figure 2), the elevation in AST and ALT levels in HCD-fed rats was significantly reduced by the oral administration of single and mixed cholesterol-lowering probiotics. Considering AST/ALT ratio, HCD-fed rats supplemented with cholesterol-lowering probiotic strains exhibited a high ratio similar to normal-diet rats, whereas the lowest ratio was observed in HCD-fed rats. The reduction of AST/ALT ratio indicated severe injury to hepatic cells due to cholesterol accumulation [26]. Thus, oral administration of single and mixed cholesterol-lowering probiotic strains could effectively relieve the liver injury induced by HCD administration. These results were consistent with the result of liver histology where cytoplasmic vacuoles with lipid deposition in livers of HCD-fed rats were observed [27]. In contrast, the reducing degrees of hepatic steatosis were observed in HCD-fed rats supplemented with cholesterol-lowering probiotic strains (Figure 3). After tissue injury, fibrosis is initiated by modifying the structure of the extracellular matrix, especially collagen fibers. Thus, Masson’s trichome staining was used to determine the progressive fibrosis after liver injury. Aggregation of collagen fibers was evidently detected in the pericentral and periportal of rats in HCD group but not in the control, probiotics, and HCD-fed rats supplemented with probiotic groups (Figure 4). These results were similar to the result of H&E staining. In addition, liver fibrosis, which is induced by HCD administration, was mitigated in HCD-rats supplemented with cholesterol-lowering probiotic strains. Fat accumulation can cause the inflammation by stimulating pro-inflammatory cytokine secretion. There are several evidences elucidating that M1-macrophages (bone marrow-derived macrophages) are key immune cells that play an important role in the immune-stimulation of tissue damage. F4/80 marker, the major surface molecules of macrophages, was determined in the liver tissues using immunohistochemical staining to study the inflammatory pathogenesis. Both bone marrow-derived macrophages and liver residential macrophages (10% of the total liver cell population) can express F4/80 marker on their cell surface; however, the infiltration of M1-macrophages entering the liver can be indicated by the increase in F4/80 staining after the subtraction of the staining of residential macrophages in the liver tissues in each rat group compared with the control group. As shown in Figure 5, there was no sign of M1-macrophage infiltration in the liver tissues in all rat groups. Based on the results of lipid profiles and liver pathology, the imbalance between cholesterol absorption and catabolism is the cause of excessive deposition of cholesterol in the extraintestinal tissues, especially adipose tissue and liver [23,28]. Moreover, the long-term administration of high-fat diets induce fat accumulation and stimulate liver fibrosis [29]. Previous studies reported that *L. plantarum* DSM20174 reduced the progression of non-alcoholic fatty liver disease by gut microbiota modulation and improved lipid accumulation and adipose tissue inflammation [30]. Therefore, body weight gain, visceral organ indexes, lipid profiles, and hepatic steatosis were significantly increased after feeding rats with HCD. However, our findings demonstrated that the oral administration of cholesterol-lowering probiotic strains could protect and treat the hypercholesterolemic effect induced by HCD administration to a great extent without adverse effects. Among the individual probiotic strains, the oral administration of *L. reuteri* TF-7 exhibited the most effect in reducing abdominal fat liver weight, TC, and TG levels. These results were in agreement with our previous study that showed *L. reuteri* TF-7 (out of 17 probiotic strains) possessed the strongest BSH activity and the highest levels of cholesterol assimilation with statistical significance [15]. Moreover, the mixed probiotic strains could relatively synergize their hypocholesterolemic effect. In some cases (TG, HDL-C, AST, and ALT levels), the mixed probiotic strains did not display the positive effects over the individual probiotic strain. This phenomenon is due to the mechanism of probiotic–probiotic and probiotic–host interaction. The cell-surface and secreted substances of the constituent probiotics play an important role in these mechanisms. The study of proteomics in probiotics reveals that various bioactive peptides from probiotic cells are associated with probiotic–probiotic and probiotic–host signaling functions as well as beneficial health effects. Basically, probiotics have to demonstrate a cellular mechanism that can activate host cells through the same signal transduction pathway Thus, they are able to synergize health-promoting effects. Several secreted substances in a glycosylated form enhance the cell signaling of both probiotic–probiotic and probiotic–host interaction. Cell-surface proteins carrying LPXTG motifs also promote cell communication between probiotics and host, which contributes to offering a synergistic effect of probiotics [31]. Thus, in this study, it is possible that individual probiotic strain had a different mechanism for inducing the signal transduction pathway on host cells. Therefore, reductions in several biochemical levels in hypercholesterolemic rats were observed with supplementation of a single strain of probiotics. However, mixed strains of probiotics failed to exhibit synergistic effect on the decrease of TG, HDL-C, AST, and ALT levels. The consistent results were reported in a previous study that there were no significant differences in the TG, HDL-C, and LDL-C levels between high-fat diet rats administered with single and combined probiotic strains [32]. Overall, three cholesterol-lowering probiotic strains may contribute to the amelioration of hypercholesterolemia associated with cardiovascular disease and metabolic disorders. The hypocholesterolemic effect of probiotics has been elucidated using several mechanisms. BSH activity and cholesterol assimilation are the major mechanisms for mitigating hypercholesterolemia. When probiotics passed through the gastrointestinal tract, BSH-producing probiotics can directly hydrolyze conjugated bile salts, both TDCA and GDCA, into deconjugated bile salts and eventually hinder in lipid emulsification. Moreover, probiotics within the gastrointestinal tract can assimilate cholesterol molecules and incorporate them in the plasma membrane. As a consequence, these mechanisms contribute in reducing hypercholesterolemia. Our results were in agreement with the previous studies reporting that oral administration of *Lactobacillus*, *Bifidobacterium*, and *Enterococcus* species exhibited relatively high therapeutic effect in reducing hypercholesterolemic diet-induced cardiovascular disease by BSH activity and cholesterol assimilation [28,33,34].

Previous studies have revealed that oral administration of probiotics could improve the gastrointestinal microbiota in order to exert health-promoting effects [35]. Thus, the changes in fecal microbiota were determined to prove this hypothesis. The present study showed that oral administration of single and mixed cholesterol-lowering probiotic strains modulated the gastrointestinal microbiota proportional to the increase in the amount of probiotics (Figure 6). In addition, these cholesterol-lowering probiotics also exhibited antimicrobial activity against pathogenic bacteria, which was similar to the result of *L. plantarum* Lp3 that showed the ability to ameliorate dysbiosis of gastrointestinal microbiota [17]. *Lactobacillus* and *Bifidobacterium* were negatively correlated with obesity [36]. *Akkermansia* can inhibit atherosclerotic lesion formation and decrease circulating endotoxin levels through prevention of metabolic endotoxemia-induced inflammation in circulation and local vascular lesions [37]. In previous studies, a reduction in the abundance of *Bacteroides* was found in atherosclerotic cardiovascular patients [38] and post-inflammation irritable bowel syndrome rats [39]. Importantly, the combination therapy of lactobacillus reversed changes in several of the above genera. This result indicated that three cholesterol-lowering probiotic strains successfully survived in the gastrointestinal tract to colonize and establish in the gastrointestinal microbiota.

## 5. Conclusions

In summary, this result indicated that the three cholesterol-lowering probiotic strains are good supplements to prevent metabolic disorders, liver injury, and cardiovascular disease. Furthermore, administration with mixed cholesterol-lowering probiotic strains causes a synergistic hypocholesterolemic effect. Overall, these findings suggest that these cholesterol-lowering probiotic strains can be developed as probiotic functional foods.

## Figures and Tables

**Table 1:** Body weight gain of rats.

Groups	Body Weight Gain (g)							
	Week 1	Week 2	Week 3	Week 4	Week 5	Week 6	Week 7	Week 8
Control	61.60 ± 17.34 ^aA^	125.00 ± 19.70 ^aB^	159.80 ± 13.52 ^aC^	219.00 ± 13.93 ^aD^	260.20 ± 8.29 ^aE^	316.40 ± 13.99 ^aF^	348.20 ± 12.40 ^aG^	305.25 ± 13.79 ^aH^
Probiotics	69.80 ± 12.52 ^aA^	122.00 ± 9.00 ^aB^	162.20 ± 11.76 ^abC^	233.00 ± 15.05 ^abD^	271.00 ± 13.34 ^aE^	325.20 ± 8.20 ^aF^	352.00 ± 14.68 ^aF^	315.52 ± 19.24 ^aG^
HCD	61.40 ± 21.62 ^aA^	135.40 ± 17.95 ^aB^	183.40 ± 4.98 ^bC^	251.80 ± 9.52 ^bD^	292.40 ± 14.74 ^cE^	350.00 ± 11.77 ^bF^	393.00 ± 15.02 ^bG^	408.22 ± 20.14 ^cG^
HCD-L	76.60 ± 33.58 ^aA^	138.80 ± 26.95 ^aB^	179.80 ± 16.45 ^acB^	239.00 ± 26.43 ^abC^	286.60 ± 13.37 ^acD^	338.40 ± 15.01 ^abE^	370.40 ± 23.93 ^abE^	361.18 ± 8.85 ^bE^
HCD-E	60.80 ± 23.33 ^aA^	122.60 ± 16.20 ^aB^	161.60 ± 19.30 ^abC^	236.80 ± 4.82 ^abD^	269.60 ± 12.50 ^acD^	334.60 ± 6.19 ^abE^	356.40 ± 17.13 ^aE^	354.41 ± 21.25 ^bE^
HCD-B	70.00 ± 4.90 ^aA^	136.20 ± 22.16 ^aB^	174.60 ± 21.87 ^abC^	236.00 ± 19.94 ^abD^	268.20 ± 22.42 ^acD^	335.20 ± 14.96 ^abE^	354.20 ± 14.31 ^aE^	364.87 ± 15.18 ^bE^
HCD-mix	62.00 ± 16.63 ^aA^	122.80 ± 11.95 ^aB^	162.40 ± 19.36 ^abC^	238.00 ± 14.68 ^abD^	264.20 ± 7.12 ^abD^	329.70 ± 9.54 ^abE^	351.20 ± 15.22 ^aE^	333.99 ± 16.19 ^aE^

The different lowercase superscript letters within the same column and uppercase superscript letters within the same row represent the statistical significance (*p* < 0.05). (n = 5, means ± SD).

**Table 2:** Food intake of rats.

Groups	Food Intake (g)							
	Week 1	Week 2	Week 3	Week 4	Week 5	Week 6	Week 7	Week 8
Control	143.68 ± 3.32 ^aA^	287.36 ± 6.63 ^aB^	431.05 ± 9.95 ^aC^	578.37 ± 17.84 ^aD^	728.91 ± 28.96 ^aE^	862.09 ± 19.89 ^aF^	1033.70 ± 59.78 ^aG^	1149.46 ± 26.52 ^aH^
Probiotics	145.98 ± 4.60 ^aA^	291.96 ± 9.20 ^aB^	437.93 ± 13.80 ^aC^	583.91 ± 18.41 ^aD^	729.89 ± 23.01 ^aE^	875.87 ± 27.61 ^aF^	1032.82 ± 27.82 ^aG^	1167.82 ± 36.81 ^aH^
HCD	148.01 ± 2.86 ^aA^	296.02 ± 5.73 ^aB^	465.32 ± 21.51 ^aC^	602.84 ± 28.07 ^aD^	753.69 ± 28.86 ^aE^	907.12 ± 35.08 ^aF^	1044.68 ± 22.47 ^aG^	1184.06 ± 22.90 ^aH^
HCD-L	146.73 ± 8.13 ^aA^	293.47 ± 16.25 ^aB^	440.20 ± 24.38 ^aC^	586.94 ± 32.50 ^aD^	733.67 ± 40.63 ^aE^	880.40 ± 48.76 ^aF^	1014.40 ± 51.44 ^aG^	1173.87 ± 65.01 ^aH^
HCD-E	144.45 ± 5.25 ^aA^	288.90 ± 10.50 ^aB^	433.36 ± 15.75 ^aC^	582.12 ± 23.33 ^aD^	722.26 ± 26.25 ^aE^	866.71 ± 31.50 ^aF^	1011.16 ± 36.75 ^aG^	1155.62 ± 42.01 ^aH^
HCD-B	145.40 ± 5.51 ^aA^	290.81 ± 11.02 ^aB^	436.21 ± 16.53 ^aC^	581.62 ± 22.04 ^aD^	729.33 ± 28.06 ^aE^	891.16 ± 33.51 ^aF^	1017.83 ± 38.58 ^aG^	1163.23 ± 44.09 ^aH^
HCD-mix	144.05 ± 6.39 ^aA^	288.09 ± 12.79 ^aB^	432.14 ± 19.18 ^aC^	578.03 ± 23.83 ^aD^	720.23 ± 31.97 ^aE^	864.28 ± 38.36 ^aF^	1011.56 ± 41.71 ^aG^	1152.37 ± 51.15 ^aH^

The different lowercase superscript letters within the same column and uppercase superscript letters within the same row represent the statistical significance (*p* < 0.05). (n = 5, means ± SD).

**Table 3:** Food efficiency of rats.

Groups	Food Efficiency (%)							
	Week 1	Week 2	Week 3	Week 4	Week 5	Week 6	Week 7	Week 8
Control	42.88 ± 12.31 ^aA^	43.48 ± 6.68 ^aA^	37.04 ± 2.46 ^aAB^	37.85 ± 1.75 ^aA^	35.72 ± 0.99 ^aAB^	36.71 ± 1.57 ^aAB^	33.76 ± 2.09 ^aAB^	26.55 ± 0.75 ^aB^
Probiotics	47.97 ± 9.46 ^aA^	41.86 ± 3.89 ^aA^	37.08 ± 3.13 ^aB^	39.94 ± 2.97 ^aA^	37.14 ± 1.82 ^aB^	37.15 ± 1.07 ^aB^	34.13 ± 2.24 ^aBC^	27.03 ± 1.64 ^abC^
HCD	41.44 ± 14.48 ^aA^	45.73 ± 5.89 ^aA^	39.82 ± 4.68 ^aA^	41.79 ± 1.14 ^aA^	38.82 ± 1.94 ^aA^	38.60 ± 1.22 ^aA^	37.64 ± 1.74 ^aA^	34.48 ± 1.59 ^cA^
HCD-L	51.97 ± 22.15 ^aA^	43.25 ± 8.43 ^aAB^	38.94 ± 4.44 ^aAB^	40.52 ± 5.01 ^aAB^	37.14 ± 2.49 ^aAB^	38.26 ± 3.15 ^aAB^	36.58 ± 2.87 ^aAB^	30.87 ± 2.27 ^cdB^
HCD-E	41.88 ± 15.29 ^aA^	42.52 ± 6.17 ^aA^	37.42 ± 5.38 ^aA^	40.72 ± 1.40 ^aA^	37.36 ± 2.04 ^aA^	37.49 ± 1.46 ^aA^	35.30 ± 2.38 ^aA^	30.73 ± 2.64 ^bcdA^
HCD-B	48.19 ± 3.75 ^aA^	44.76 ± 6.50 ^aA^	40.08 ± 5.31 ^aB^	42.32 ± 3.73 ^aA^	37.87 ± 2.69 ^aBC^	37.74 ± 3.00 ^aBC^	34.83 ± 1.69 ^aBC^	31.40 ± 1.75 ^cdC^
HCD-mix	42.78 ± 10.03 ^aA^	42.70 ± 4.62 ^aA^	37.62 ± 4.56 ^aAB^	40.04 ± 3.33 ^aA^	38.13 ± 1.92 ^aAB^	37.90 ± 2.06 ^aAB^	35.56 ± 2.41 ^aAB^	29.02 ± 1.86 ^abdB^

The different lowercase superscript letters within the same column and uppercase superscript letters within the same row represent the statistical significance (*p* < 0.05). (n = 5, means ± SD).

**Table 4:** Abdominal fat, abdominal fat index, liver weight, and liver index of rats.

Groups.	Parameters			
	Abdominal Fat (g)	Abdominal Fat Index (mg/g Body Weight)	Liver Weight (g)	Liver Index (mg/g Body Weight)
Control	9.90 ± 0.56 ^a^	22.65 ± 1.53 ^a^	10.81 ± 0.72 ^a^	24.71 ± 1.60 ^a^
Probiotics	9.93 ± 0.92 ^a^	22.21 ± 2.87 ^a^	11.07 ± 0.97 ^a^	24.62 ± 1.46 ^a^
HCD	19.06 ± 2.30 ^b^	35.08 ± 4.89 ^b^	18.22 ± 1.01 ^b^	33.54 ± 2.92 ^b^
HCD-L	13.57 ± 1.92 ^c^	28.01 ± 4.66 ^ab^	14.31 ± 3.13 ^c^	29.45 ± 6.42 ^ab^
HCD-E	13.99 ± 1.91 ^c^	28.47 ± 3.59 ^ab^	14.35 ± 1.18 ^c^	29.25 ± 2.70 ^ab^
HCD-B	13.99 ± 1.24 ^c^	28.10 ± 2.31 ^ab^	14.40 ± 0.63 ^c^	28.95 ± 1.44 ^ab^
HCD-mix	11.89 ± 1.74 ^ac^	25.28 ± 3.79 ^a^	11.38 ± 1.85 ^ac^	24.14 ± 3.62 ^a^

The different lowercase superscript letters within the same column represent the statistical significance (*p* < 0.05). (n = 5, means ± SD).

## Data Availability

The data are contained within this article.
